# Psychometric development of a moral distress scale for healthcare education and practice

**DOI:** 10.3389/fpsyg.2025.1661414

**Published:** 2025-09-26

**Authors:** Hung-Chang Liao, Ya-Huei Wang

**Affiliations:** ^1^Department of Health Policy and Management, Chung Shan Medical University, Taichung, Taiwan; ^2^Department of Medical Management, Chung Shan Medical University Hospital, Taichung, Taiwan; ^3^Department of Applied Foreign Languages, Chung Shan Medical University, Taichung, Taiwan; ^4^Department of Medical Humanities, School of Medicine, Chung Shan Medical University, Taichung, Taiwan; ^5^Department of Medical Education, Chung Shan Medical University Hospital, Taichung, Taiwan

**Keywords:** moral distress, scale development, healthcare education, psychometric validation, factor analysis

## Abstract

**Objective:**

Unaddressed moral distress may result in psychological, emotional, and physical consequences. The study was to develop and validate a Moral Distress Scale for Healthcare Students and Providers (MDS-HSP) within the Taiwanese healthcare education and clinical contexts, providing a framework for administrators and policymakers to recognize and respond to moral distress in training and practice settings.

**Methods:**

Following an extensive literature review and expert discussions, the study performed an exploratory factor analysis (EFA) using SPSS with a sample of 332 participants to determine the hidden structure of the MDS-HSP and evaluate its initial psychometric properties. A subsequent confirmatory factor analysis (CFA) using AMOS with a separate sample of 240 participants was performed to verify the identified factor structure. The testing process included the assessments of validity, reliability, and goodness-of-fit analysis.

**Results:**

Following the EFA, the initial 72 items were refined to 42 items across six factors: “acquiescence to patients’ rights violations” (8 items), “lack of professional competence” (9 items), “disrespect for patients’ autonomy” (10 items), “futile treatment” (5 items), “organizational and social climate” (6 items), and “not in patients’ best interest” (4 items). The CFA confirmed the same six scale factors and 42 items. Both EFA and CFA supported the proposed factor structure and demonstrated adequate validity and reliability.

**Conclusion:**

The study provided empirical evidence supporting the MDS-HSP as a reliable tool for assessing moral distress experienced by healthcare students and providers. Its use may inform educational strategies, institutional policies, and ethical support mechanisms within healthcare and academic settings.

## 1 Introduction

As medical technology and information continue to advance rapidly, healthcare professionals are increasingly confronted with complex caregiving demands, often within the constraints of limited resources and the pressure for cost-efficiency. In such high-stakes environments, ethical ideals often collide with the complexities of real-world clinical environments (Topol, 2013). However, many institutions still lack standardized ethical guidelines to navigate these challenges, leaving caregivers feeling overwhelmed, helpless, and demoralized. As a result, healthcare providers often find themselves disoriented and emotionally distressed when making decisions in morally ambiguous situations (Austin et al., 2017; Hamric and Blackhall, 2007).

Jameton (1984) first introduced moral distress and described it as the psychological discomfort of knowing the morally appropriate course of action to take but failing to act on it because of external constraints. In examining the impacts of moral distress on neonatal intensive care unit nurses, Jameton (1984) highlighted how institutional or systemic barriers can hinder nurses from delivering optimal care but feel compelled to carry out actions they consider morally wrong. The definition of moral distress has been expanded over time to encompass a wider range of ethical challenges, including the psychological and physical tolls that result from such distress. Moral distress emerges when individuals are forced to act against their moral beliefs, preventing them from upholding their core values and resulting in a sense of powerlessness to alter the situation (Godshall, 2021; Jameton, 1993; Salari et al., 2022). In healthcare, moral distress arises primarily when professionals must administer treatments that they know are ineffective or do not serve the patient’s best interests, especially true in circumstances of futile medical care (Rice et al., 2008). This type of distress is prevalent among nurses working in high-pressure settings such as acute care units, obstetrics, pediatric wards, and acute psychiatric departments (Ferrell, 2006; Jansen et al., 2020). Compared to other healthcare professionals, nurses tend to experience moral distress both more frequently and intensely (Corley, 2002; Hamric and Blackhall, 2007; Sporrong et al., 2006).

Moral distress impacts not only nurses but also other healthcare providers, including doctors, physiotherapists, occupational therapists, speech therapists, pharmacists, dietitians, etc., (Brazil et al., 2010; Hamric, 2010; Ulrich and Grady, 2018). It presents a significant challenge for healthcare providers, particularly those working in high-pressure environments, such as emergency medical units, intensive care units, etc., (Lamiani et al., 2017), where they are frequently exposed to moral distress and psychological strain, especially during the pandemic. A meta-analysis of moral distress revealed that it is more frequently experienced by providers when they feel they are administering excessive care. It is less common when palliative care options are suggested (Prentice et al., 2016). ICU healthcare providers are more likely to suffer moral distress when faced with end-of-life situations, ethical dilemmas, and complicated family dynamics (Coughlin, 2021). Whitehead et al. (2015) noted that moral distress in ICU settings often comes from a lack of consistent care, pressure to follow family requests that conflict with the patient’s best interests, and adverse effects of ineffective communication. Additional challenges involve administering treatments that may be deemed inappropriate or ineffective, making life-or-death decisions, and withholding information from patients or their families–all of which can intensify moral distress (Corley, 1995; Ferrell, 2006; Fujii et al., 2021). Some other factors that may cause moral distress are poor teamwork, working with incompetent colleagues, fear of judgment from colleagues, improper allocation of medical resources, shortages of staff and resources, the continuation of treatments that merely prolong suffering, and the use of interventions deemed futile, which can cause unnecessary pain to the patient (Al-Humadi et al., 2021; Beltrão et al., 2023; Haghighinezhad et al., 2019).

If left unaddressed, moral distress may bring in a range of adverse emotional and psychological outcomes, such as anger, anxiety, shame, guilt, sadness, frustration, emotional numbness, cynicism, or self-criticism (Burston and Tuckett, 2012; Jameton, 1984, 2017; Jansen et al., 2020; Parker and Tavella, 2021; Rushton, 2018). Furthermore, they may emotionally disengage from their patients and distance themselves from others (Lamiani et al., 2017; Wilson et al., 2013). Physically, they may suffer from burnout, compassion fatigue, headaches, stomach issues, sleep disturbances, weight changes, palpitations, and medical errors (Delfrate et al., 2018; Rushton, 2018). Ultimately, unresolved moral distress may undermine healthcare quality, diminish patient satisfaction, and increase staff turnover (Burston and Tuckett, 2012; Huffman and Rittenmeyer, 2012; McCarthy and Gastmans, 2015).

Addressing the detrimental impacts of moral distress on healthcare personnel, organizations, and the overall healthcare system is an urgent concern (Aultman and Wurzel, 2014; Musto et al., 2015). Therefore, developing tools for early identification and accurate assessment of moral distress is essential, as this provides a foundation for designing effective intervention strategies (Lachman, 2016). Corley et al.’s (2001) Moral Distress Scale (MDS) is the pioneering instrument developed to measure moral distress, grounded in Jameton’s (1984) original conceptualization. The 38-item scale was initially developed to assess how often and severely nurses, especially those working in the ICU, experience moral distress (Corley et al., 2001). Despite its widespread use, the original MDS demonstrated acceptable internal consistency but lacked confirmatory factor analysis and was validated only in nurses. Hamric et al. (2012) modified the tool to 21 questions to assess moral distress. Today, the revised tool, MDS-Revised, remains the commonly employed measure to assess how often and how severely healthcare providers experience moral distress in diverse hospital settings (Hamric and Epstein, 2017). The MDS-R reduced the number of items but retained similar limitations regarding construct validity and generalizability. Different versions of the scale have been adapted to fit the unique needs of various healthcare practitioners, including Wocial and Weaver’s (2013) Moral Distress Thermometer (MDT) and Epstein et al.’s (2019) Measure of Moral Distress for Healthcare Practitioners (MMD-HP). These versions have shown robust validity and reliability across diverse healthcare professions but may require cultural adaptation.

Moreover, several challenges may emerge when considering direct adaptation of the MDS-R or MMD-HP into the Taiwanese context. First, moral distress varies depending on each individual’s cultural background and the unique circumstances of the healthcare setting (Horton et al., 2007). Additionally, the moral distress and ethical dilemmas embedded in these tools reflect Western healthcare systems and do not sufficiently resonate with the socio-cultural dynamics of the Taiwanese healthcare system, especially issues such as family authority and role-based power imbalances (Yeh et al., 2010). Given that environmental and cultural factors influence the experience and response to moral distress (Hamric et al., 2012; Tian et al., 2021), existing tools might not completely reflect the unique moral distress experienced by healthcare students and providers in Taiwanese culturally specific healthcare environments. Moreover, linguistic equivalence alone is insufficient to convey culturally embedded concepts, such as filial piety and collective decision-making, which profoundly shape clinical ethics in Taiwan (Yeh et al., 2010). These systemic, cultural, and linguistic differences make direct adaptation problematic and therefore justify the development of a culture-specific instrument (Picconi et al., 2023).

Hence, without cultural adaptation and validation, the use of these tools in Taiwan may result in incomplete or misleading assessments of moral distress. Consequently, to address this gap, the study intended to go through a systematic review to develop and validate a scale (MDS-HSP) for assessing moral distress in this population based on Taiwanese cultural contexts.

## 2 Methodology

### 2.1 Procedure and participants

Grounded in an extensive literature review on moral distress, ethical tensions, moral dilemmas, and related psychological distress, the researchers initially identified 83 potential items capturing various facets of moral distress. To refine this item pool, a series of expert panel discussions (Boateng et al., 2018) was conducted with three professionals specializing in psychometrics, medical humanities, and medical education. Each expert independently evaluated the items using a 6-point relevance scale (0: not relevant; 5: extremely relevant). Items scoring below 4 or lacking inter-rater agreement were eliminated from further consideration. This process constituted a formal evaluation of content validity by the expert panel prior to item testing. Through iterative discussions and consensus-building, the item set was streamlined to 72 items. A 9-point Likert scale was adopted, where 1 represented the absence of distress and 9 denoted the highest level of moral distress, with higher scores reflecting more intense experiences of moral distress.

The researchers carried out a pilot study, employing exploratory factor analysis (EFA) on data collected from 332 participants comprising medical and healthcare students and providers. To validate the identified factor structure, confirmatory factor analysis (CFA) was subsequently carried out on an independent sample of 240 participants. Participants were healthcare students and providers in Taiwan aged 18 or older who completed the survey; those under 18, outside healthcare roles, or with incomplete responses were excluded. The Institutional Review Board of Chung Shan Medical University Hospital granted approval for the research (IRB No. 112008).

### 2.2 Data analysis

The study first evaluated the normality of the data using skewness and kurtosis. According to Hair et al. (2018), skewness and kurtosis values within ±2.58 (*p* < 0.01) or ±1.96 (*p* < 0.05) are generally considered indicative of a normal distribution. Byrne (2010) further noted that kurtosis values of 7 or higher suggest a deviation from normality. Kline (2016) suggested that an absolute skewness value exceeding 3.0 (|γ_1_| > 3.0) indicates severe skewness, and an absolute kurtosis value exceeding 10.0 (|γ_2_| > 10.0) indicates a potential problem. To uncover and confirm the underlying factor structure, the study conducted an EFA using SPSS version 14.0 (IBM Corp, 2016) on data from 332 participants, followed by CFA with AMOS version 24.0 (Arbuckle, 2016) on a separate sample of 240 individuals. Factor extraction was guided by eigenvalue assessment, principal component analysis (PCA), and Promax rotation to accommodate potential correlations among factors. Sampling adequacy and the acceptability of data for factor analysis were assessed through the Kaiser-Meyer-Olkin (KMO; Kaiser, 1970; Kaiser and Rice, 1974) and Bartlett’s test of sphericity (Bartlett, 1950, 1951). Model fit was assessed for both EFA and CFA using a range of statistical indicators, including the chi-square to degrees of freedom ratio (χ^2^/*df*; Hooper et al., 2008), Tucker-Lewis Index (TLI; Bentler, 1990), Comparative Fit Index (CFI; Bentler, 1990), and the Root Mean Square Error of Approximation (RMSEA; Hooper et al., 2008). Furthermore, the psychometric evaluation of the scale included tests for convergent and discriminant validity, along with reliability analysis using composite alpha and Cronbach’s alpha.

## 3 Results

### 3.1 Preliminary data analysis and suitability for factor analysis

**FIGURE 1:**
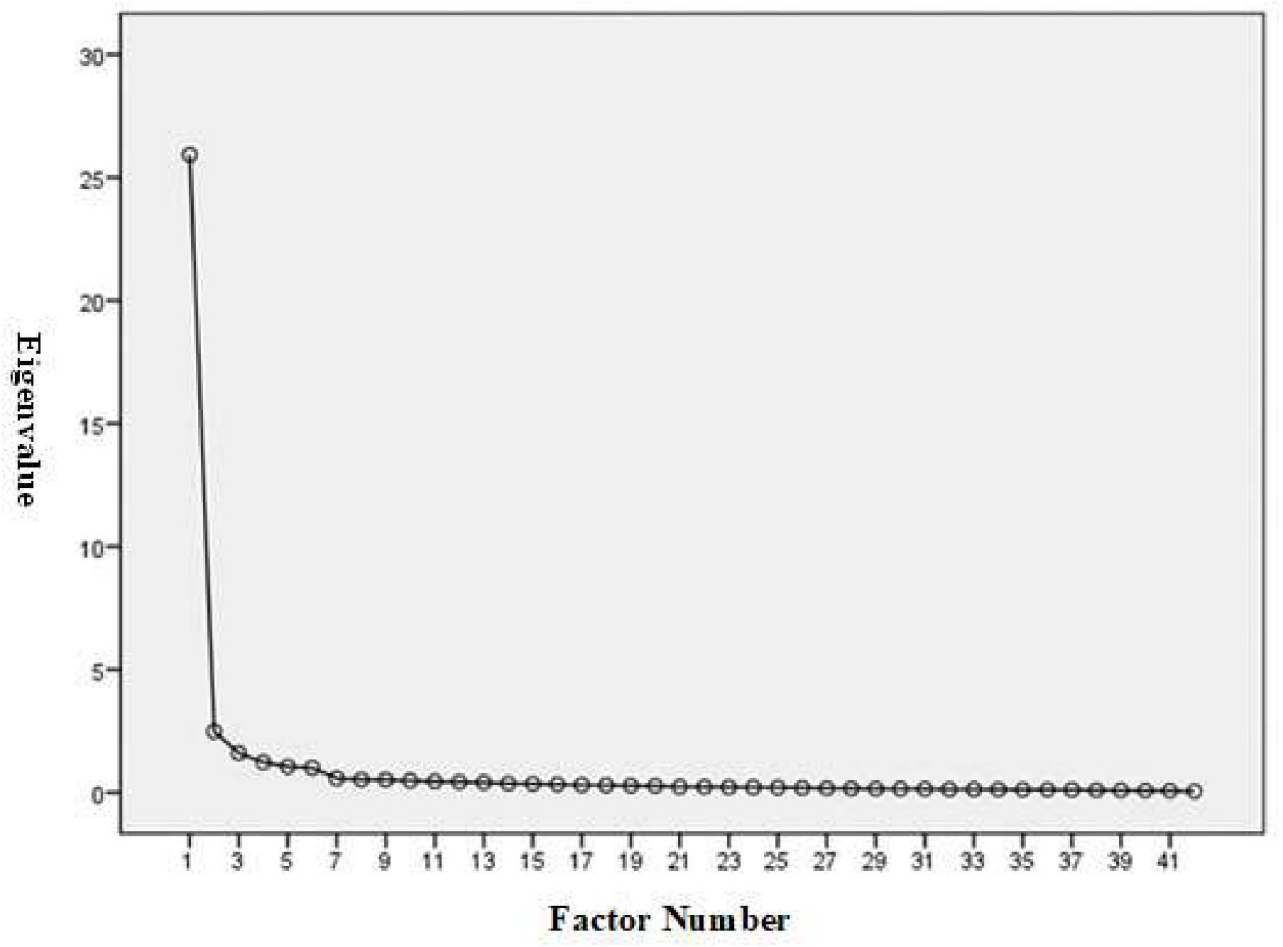
Scree plot for factor analysis of the MDS-HSP.

A total of 332 completed questionnaires were obtained from healthcare students and providers across Taiwan. The researchers assessed outliers and multivariate normality by examining skewness and kurtosis. Preliminary analysis revealed no extreme values, with skewness ranging within ±1 and kurtosis within ±2, indicating adequate normality. To assess whether the dataset was suitable for EFA, two statistical tests were conducted. The KMO yielded an exceptionally high value of 0.975, well above the accepted criterion of 0.6 (Kaiser, 1970; Kaiser and Rice, 1974), indicating excellent sampling adequacy. Bartlett’s test of sphericity also yielded a highly significant result (Approx. = 16,704.881; *df* = 861; *p* < 0.001), indicating that the factors were appropriately correlated for factor analysis (Bartlett, 1950, 1951). These outcomes validated the appropriateness of proceeding with factor extraction. The scree plot analysis for the MDS-HSP instrument indicated that a six-factor solution is the most appropriate structural representation of the data (Figure 1).

Among the 332 participants, 77 participants (23.2%) were male, and 255 participants (76.8%) were female. There were 2 participants (0.6%) who were below the age of 19, 46 participants (13.8%) who were between the ages of 19 and 25, 132 participants (39.8%) who were between the ages of 26 and 32, 90 participants (27.1%) who were between the ages of 33 and 39, 48 participants (14.5%) who were between the ages of 40 and 46, and 13 participants (3.9%) who were between the age of 47 and 53, 1 participant (0.3%) who was between the ages of 54 and 60, and 0 participant (0%) who was 61 years old or older. Healthcare students comprised 188 (56.6%) of these participants, while healthcare providers comprised 144 (43.4%). The participants’ demographic details are shown in Table 1.

**TABLE 1:** Participants’ demographic details.

Condition	Categories	Number	Percentage
Gender	Male	77	23.2
	Female	255	76.8
Age	19 years old	2	0.6%
	19+ to 25 years old	46	13.8%
	26+ to 32 years old	132	39.8%
	33+ to 39 years old	90	27.1%
	40+ to 46 years old	48	14.5%
	47+ to 53 years old	13	3.9%
	54+ to 60 years old	1	0.3%
	61+ years old	0	0%
Source	Health students	188	56.6%
Healthcare providers	144	43.4%

### 3.2 Exploratory factor analysis (EFA)

To examine the MDS-HSP scale’s construct and internal consistency validity, the researchers employed EFA, using PCA with Promax rotation and an eigenvalue threshold of 1.0. Items were retained if they demonstrated a loading of ≥0.50 on their designated factor and <0.50 on unrelated factors. This analysis identified six distinct factors encompassing a total of 42 items, collectively accounting for 79.268% of the total variance. The first factor, “acquiescence to patients’ rights violations,” comprised 8 items and explained the largest portion of variance at 61.727%. The second factor, “lack of professional competence,” included 9 items and contributed 5.910%. The third, “disrespect for patients’ autonomy,” encompassed 10 items and accounted for 3.812% of the variance. The fourth factor, “futile treatment,” consisted of 5 items and explained 2.927%. The fifth, “organizational and social climate,” was composed of 6 items and contributed 2.506%, while the sixth and final factor, “not in patients’ best interest,” contained 4 items and explained 2.387%. Each factor exhibited an eigenvalue exceeding the threshold of 1.0, specifically: 25.925, 2.482, 1.601, 1.229, 1.052, and 1.003, thereby confirming the statistical significance and multidimensional nature of the scale structure (see Table 2).

**TABLE 2:** Rotated factor loading and Cronbach’s alpha for the MDS-HSP scale.

Item	Factor 1	Factor 2	Factor 3	Factor 4	Factor 5	Factor 6
**Factor 1: α = 0.968**						
52	0.961	0.008	0.035	−0.042	−0.044	−0.014
56	0.935	0.078	−0.084	0.027	−0.079	0.042
53	0.932	−0.040	0.051	−0.029	−0.029	0.041
54	0.854	0.014	0.098	−0.038	0.049	−0.113
57	0.819	0.059	0.012	−0.043	0.109	−0.042
58	0.817	0.018	−0.098	0.058	0.155	−0.009
51	0.775	0.063	0.026	0.061	0.031	0.019
49	0.696	−0.037	0.097	0.127	−0.018	0.063
**Factor 2: α = 0.958**						
3	0.061	0.887	0.098	−0.133	−0.050	0.040
6	−0.017	0.848	−0.010	0.015	0.038	0.081
5	0.099	0.813	0.123	−0.075	−0.091	0.077
2	0.011	0.789	0.009	0.093	0.029	−0.111
7	−0.158	0.743	−0.045	0.108	0.278	0.026
4	−0.021	0.671	0.246	−0.023	0.132	−0.038
11	0.103	0.655	−0.149	0.129	−0.114	0.247
8	0.062	0.644	0.223	0.007	0.017	−0.011
1	0.012	0.602	0.463	−0.076	−0.024	−0.115
**Factor 3: α = 0.972**						
28	−0.029	0.034	0.895	0.023	−0.027	0.007
27	−0.0218	−0.069	0.864	0.094	0.104	0.097
29	0.092	0.084	0.834	−0.113	0.000	0.056
30	0.135	0.150	0.803	−0.076	−0.009	−0.066
31	0.101	0.049	0.760	−0.016	0.104	−0.031
23	0.130	0.133	0.743	0.101	−0.154	−0.055
21	0.018	0.128	0.741	0.093	0.012	−0.065
20	0.076	0.198	0.717	0.108	−0.062	−0.108
22	0.099	0.128	0.694	0.134	−0.094	−0.011
32	0.120	−0.023	0.547	−0.026	0.096	0.325
**Factor 4: α = 0.890**						
47	−0.126	−0.145	0.254	0.874	0.000	−0.018
46	0.033	0.017	0.103	0.806	−0.035	0.007
44	−0.035	0.153	−0.123	0.730	0.237	−0.110
45	0.161	0.061	−0.066	0.722	−0.081	0.057
48	0.085	0.014	0.040	0.659	−0.031	0.130
**Factor 5: α = 0.954**						
63	−0.023	0.014	−0.052	0.038	0.917	0.044
64	−0.010	0.104	−0.155	−0.013	0.892	0.165
65	0.129	0.024	0.126	−0.023	0.758	−0.102
67	0.249	−0.080	0.214	−0.041	0.568	0.038
69	0.242	0.013	0.180	0.060	0.557	−0.085
68	0.168	0.025	0.272	0.023	0.542	−0.041
**Factor 6: α = 0.867**						
35	−0.002	0.106	−0.358	0.084	0.011	0.932
33	−0.094	0.028	0.168	−0.087	0.027	0.879
36	0.052	−0.002	0.205	−0.053	0.139	0.690
34	0.110	−0.164	0.376	0.132	−0.048	0.527
Eigen value	25.925	2.482	1.601	1.229	1.052	1.003
% of variance	61.727	5.910	3.812	2.927	2.506	2.387

SD, standard deviation. Overall α = 0.984; total variation explained: 79.268%.

#### 3.2.1 Validities and reliability of the MDS-HSP scale

First written in English, the MDS-HSP scale was later translated into Chinese. To ensure linguistic accuracy and conceptual equivalence, a bilingual expert later back-translated the version into English, allowing for comparison with the initial draft. Content validity was further established through expert evaluation. Additionally, three university students were involved in refining the wording of certain items to improve clarity and ease of understanding.

Cronbach’s alpha coefficients were calculated to assess the internal consistency of the MDS-HSP scale. According to Churchill (1979), a value above 0.8 is generally considered ideal, while 0.7 marks the threshold for acceptable reliability. The overall Cronbach’s alpha for the full scale was 0.984, proving exceptional internal reliability. The analysis also revealed high reliability across all six subscales, with alpha values of 0.968, 0.958, 0.972, 0.890, 0.954, and 0.867, respectively. The results affirmed that both the individual factors and the entire MDS-HSP scale provided stable and consistent measurement of moral distress among participants (Table 2).

#### 3.2.2 Descriptive statistics for the eFA-model MDS-HSP scale

Table 3 shows descriptive statistics for the MDS-HSP scale’s six factors, including item descriptions, mean scores (*M*), and standard deviations (SD).

**TABLE 3:** Item descriptions, mean scores (*M*), and standard deviations (SD) on the MDS-HSP scale.

Item	*M*	SD
Factor 1. Acquiescence to patients’ rights violations	36.798	19.336
52. Daring not to take any action but remaining silent when noting that healthcare staff do not honestly report patients’ deaths brought on by their improper treatment or misdiagnosis	4.71	2.831
56. Daring not to take any action but remaining silent when noting that healthcare staff do not provide patients/guardians with sufficient information to ensure their informed consent	4.62	2.597
53. Daring not to take any action but remaining silent when noting that terminally-ill patients are being abandoned by their families	4.58	2.666
54. Daring not to take any action but remaining silent when noting that patients might be victims of abuse or violence	4.31	2.776
57. Daring not to take any action but remaining silent when witnessing healthcare staff make fun of patients	4.42	2.662
58. Daring not to speak up for patients but remaining silent when being requested to administer a range of life-sustaining treatments, which will, in my opinion, only prolong the patients’ dying process	4.64	2.634
51. Daring not to take any action but remaining silent when noting that healthcare staff do not respect patients’ privacy	4.59	2.654
49. Remaining silent about observed unethical behavior in the workplace for fear of jeopardizing my job	4.94	2.585
Factor 2. Lack of professional competence	44.970	21.340
3. Witnessing a decline in patient care quality as a result of inadequate healthcare team communications	5.28	2.680
6. Witnessing a decline in patient care quality due to a lack of caregiver continuity	5.22	2.685
5. Witnessing healthcare staff depriving patients of needed medical care due to the arrogance and uncooperativeness of patients and their families	5.15	2.828
2. Being requested to care for patients whom I do not believe I am professionally qualified to provide such care	4.93	2.686
7. Working in situations where the number of healthcare workers is too small to provide adequate medical care	5.09	2.736
4. Working with an incompetent multidisciplinary healthcare team	4.85	2.740
11. Responding to patients’ requests for help with ending their life when they have a poor prognosis	4.87	2.641
8. Working with doctors who do not explain to their patients their health status and disease	4.84	2.727
1. Working with healthcare providers (doctors, nurses, technicians, assistants, etc.) who lack professional qualifications or provide inappropriate health services	4.74	2.960
Factor 3. Disrespect for patients’ autonomy	47.706	25.910
28. Witnessing healthcare staff accepting bribes and favored privileges	4.47	2.867
27. Witnessing healthcare staff using free health check-ups and free consultations as a tactic to attract patients for medical consumption	4.61	2.702
29. Witnessing healthcare staff favoring privileges	4.74	2.981
30. Witnessing healthcare staff disregarding patient safety for the sake of profits	4.91	3.250
31. Witnessing healthcare staff requesting informed consent from patients for innovative treatments in human trials, while the management of potential side effects remains inadequately addressed	4.76	3.039
23. Being requested to resuscitate no-code terminally ill patients under strong pressure from patient families	4.70	2.787
21. Witnessing healthcare staff taking drastic medical measures to save the lives of terminally ill patients who have clearly expressed a wish to die	4.79	2.846
20. Witnessing medical staff responsible for patient care not discussing patients’ prognosis with patients or their families, even when asked for the truth	4.92	2.912
22. Witnessing healthcare staff not discussing emergency treatment options (code status) with the families of terminally ill or comatose patients before cardiac arrest	4.78	2.772
32. Being requested to abandon critically ill patients who had a chance to survive in situations where medical resources (respirators, life support equipment, gowns, beds, and workforce) are insufficient	5.03	2.838
Factor 4. Futile treatment	23.960	10.572
47. Being requested to provide aggressive yet potentially futile surgical treatment for terminally ill patients	4.46	2.436
46. Being requested to participate in surgeries for terminally ill patients	4.62	2.447
44. Asking patients’ families to donate organs to save others when death is unavoidable	4.78	2.647
45. Being requested to perform extreme life-saving measures to save patients, which, I think, only prolongs their dying process	5.12	2.628
48. Feeling powerless yet complying with doctors’ orders to continue treatment due to insistence from patients or their families	4.98	2.516
Factor 5. Organizational and social climate	28.736	14.327
63. Providing subpar treatment or care as a result of administrative pressure or limitations in total healthcare coverage	4.99	2.539
64. Providing less than optimal care to unconscious patients due to staffing shortages	5.05	2.568
65. Providing services that are outside my field of practice due to staff shortages in other areas or specialties	4.80	2.677
67. Discontinuing treatment under institutional policies when patients cannot pay fees	4.59	2.710
69. Working in situations where necessary medical equipment or resources are lacking and the quality of medical care cannot be guaranteed	4.64	2.731
68. Working in organizations lacking the necessary equipment to provide emergency assistance to patients	4.67	2.675
Factor 6. Not in patients’ best interest	18.829	8.661
35. Following patient families’ and physicians’ decision to extubate or remove life-sustaining equipment from patients	4.80	2.620
33. In conditions of limited medical resources, following physicians’ instructions to prioritize treatment for patients with higher survival rates and longer life expectancy	4.88	2.555
36. In situations where medical resources (respirators, life support equipment, gowns, beds, and workforce) are insufficient, being requested to abandon patients who require more medical resources	4.76	2.551
34. Witnessing patients dying due to their refusal of blood transfusions based on their doctrines and beliefs	4.39	2.516

### 3.3 CFA for the MDS-HSP scale

The study further performed CFA using data from 240 participants in order to further confirm the acquired factor structure, of whom 91 participants (37.9%) were male, 140 participants (58.3%) were female, and 9 participants (3.8%) preferred not to say. There were 8 participants (3.3%) who were below the age of 19, 41 participants (17.1%) who were between the ages of 19 and 25, 110 participants (45.8%) who were between the ages of 26 and 32, 48 participants (20.0%) who were between the ages of 33 and 39, 26 participants (10.8%) who were between the ages of 40 and 46, and 6 participants (2.5%) who were between the age of 47 and 53, 0 participant (0%) who were between the ages of 54 and 60, and 1 participant (0.4%) who were 61 years old or older. Healthcare students comprised 106 (44.2%) of these participants, while healthcare providers comprised 134 (55.8%). The participants’ demographic details are shown in Table 4.

**TABLE 4:** Participants’ demographic details.

Characteristics	Categories	Number	Percentage
Gender	Male	91	37.9
	Female	140	58.3
	Prefer not to say	9	3.8
Age	19 years old	8	3.3%
	19+ to 25 years old	41	17.1%
	26+ to 32 years old	110	45.8%
	33+ to 39 years old	48	20.0%
	40+ to 46 years old	26	10.8%
	47+ to 53 years old	6	2.5%
	54+ to 60 years old	0	0%
	61+ years old	1	0.4%
Source	Health students	106	44.2
Health providers	134	55.8

**FIGURE 2:**
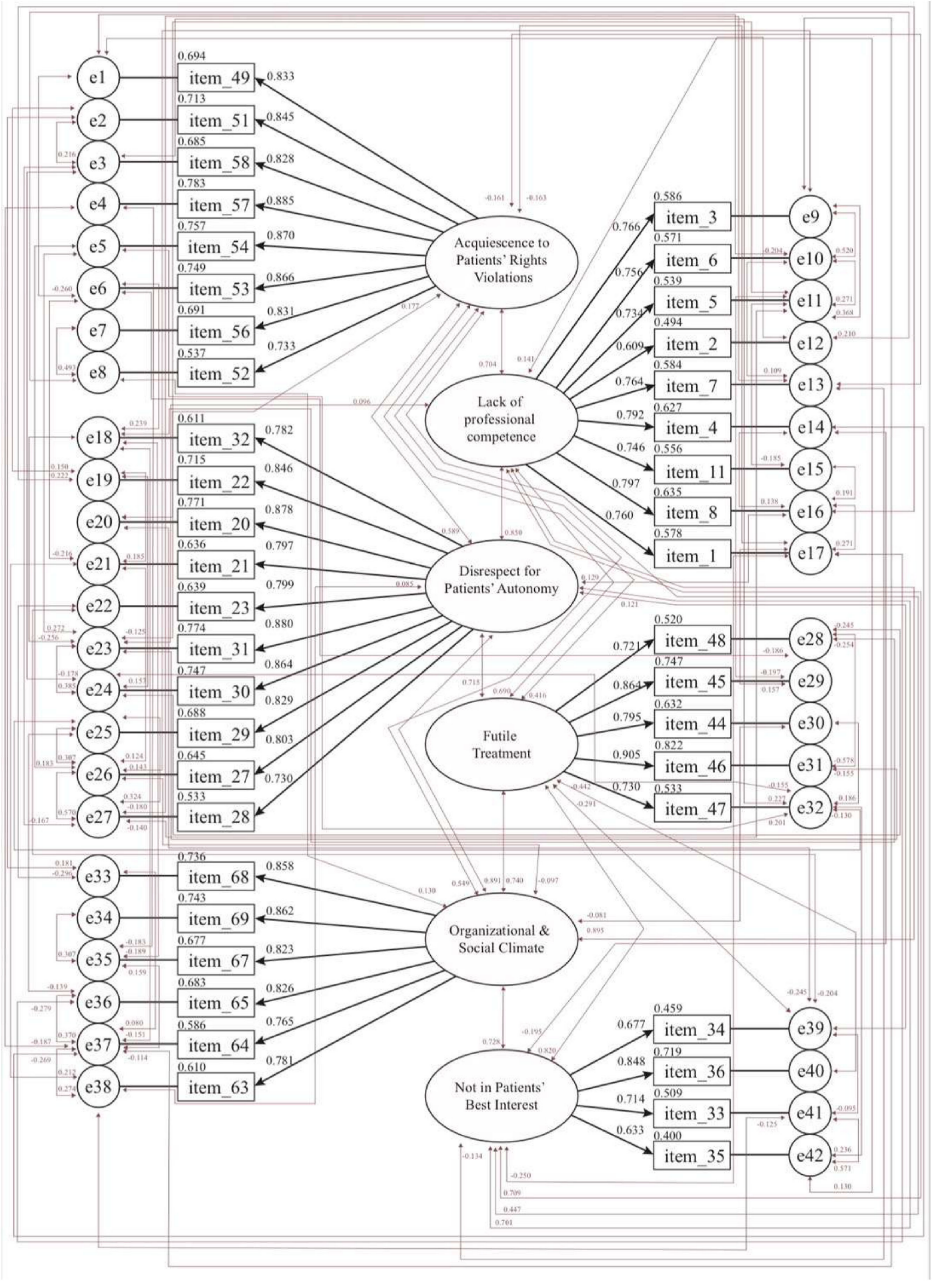
Confirmatory factor analysis (CFA) diagram for the MDS-HSP scale.

The CFA, done with the AMOS (Arbuckle, 2016), validated the same six scale factors and 42 items (Figure 2). No items were deleted from the factors of “acquiescence to patients’ rights violations” (8 items; factor loadings: 0.733–0.885), “lack of professional competence” (9 items; factor loadings: 0.609–0.797), “disrespect for patients’ autonomy” (10 items; factor loadings: 0.730–0.880), “futile treatment” (5 items; factor loadings: 0.721–0.906), “organizational and social climate” (6 items; factor loadings: 0.765–0.862), and “not in patients’ best interest” (4 items; factor loadings: 0.633–0.848), respectively.

#### 3.3.1 Goodness of fit

To thoroughly assess the suitability of both the EFA and CFA models, the study utilized a range of fit statistics. Among them, the χ^2^/*df* was adopted to gauge how closely the observed data matched the model’s expectations. A *p*-value under 0.05 indicates only a slight divergence between the actual data and the model. Nonetheless, the ideal χ^2^/*df* cutoff remains debated, with some scholars arguing that a value between 2.0 and 5.0 is acceptable (Tabachnick and Fidell, 2007; Wheaton et al., 1977), while others proposed a more rigorous standard of below 2.0 to indicate a superior fit (Koufteros, 1999; Schumacker and Lomax, 2010). Additional validation came from the TLI and CFI, with scores above 0.90 generally reflecting an acceptable fit, and values of 0.95 or higher signaling an exceptional fit (Hu and Bentler, 1999; Schermelleh-Engel and Moosbrugger, 2003; Tucker and Lewis, 1973). The RMSEA was also employed to examine how closely the model mirrored the actual data structure (Chen, 2007; Hooper et al., 2008). Hu and Bentler (1999) asserted that RMSEA values under 0.08 point to a reasonable fit, with values below 0.05 reflecting optimal superior model fit. Table 5 lists all fit indices applied in evaluating the MDS-HSP scale’s structure through both exploratory and confirmatory factor analysis. For the EFA model of the MDS-HSP, the model had a chi-square-to-degrees-of-freedom ratio (χ^2^/*df*) of 2.401, a TLI of 0.87, a CFI of 0.88, and an RMSEA of 0.08. The CFA model has a χ^2^/*df* of 1.097, a TLI of 0.99, a CFI of 0.99, and an RMSEA of 0.02, all exceeding commonly accepted thresholds (Hu and Bentler, 1999). These results provide strong support for the six-factor structure of the MDS-HSP.

**TABLE 5:** Goodness-of-fit indexes.

Model	χ^2^	df	*χ*^2^/*df*	*P*	TLI	CFI	RMSEA
EFA-model MDS-HSP	1930.23	804	2.401	0.000	0.87	0.88	0.08
CFA-model MDS-HSP	777.48	709	1.097	0.037	0.99	0.99	0.02

#### 3.3.2 Reliability and validity

To assess the reliability and consistency of the CFA-based MDS-HSP scale, the researchers analyzed both composite reliability (CR) and Cronbach’s alpha. All scores were above the 0.70 cutoff, reflecting high reliability and stable psychometric properties (Churchill, 1979; Hair et al., 2018). Specifically, CR scores for the six factors and the overall instrument fell between 0.812 and 0.987, while Cronbach’s alpha values fell between 0.832 and 0.974. These high coefficients, covering factors of “acquiescence to patients’ rights violations,” “lack of professional competence,” “disrespect for patients’ autonomy,” “futile treatment,” “organizational and social climate,” and “not in patients’ best interest” confirmed the scale’s internal consistency and reliability (see Table 6). To determine whether the MDS-HSP scale items effectively captured the underlying constructs, the study assessed convergent validity through Average Variance Extracted (AVE) and CR scores. Convergent validity is supported when the AVE exceeds 0.50, yet remains lower than the corresponding CR, which should surpass 0.60 (Hair et al., 2018; Pallant, 2013). Table 6 summarizes the AVE and CR values for each of the six identified MDS-HSP scale factors: “acquiescence to patients’ rights violations” (AVE: 0.702; composite alpha: 0.949), “lack of professional competence” (AVE: 0.561; composite alpha: 0.920), “disrespect for patients’ autonomy” (AVE: 0.676; composite alpha: 0.954), “futile treatment” (AVE: 0.650; composite alpha: 0.902), “organizational and social climate” (AVE: 0.676; composite alpha: 0.925), and “not in patients’ best interest” (AVE: 0.522; composite alpha: 0.812). Significantly, each AVE value was lower than its corresponding CR, which consistently exceeded 0.70, highlighting robust reliability across all scale factors (Hair et al., 2018; Pallant, 2013).

**TABLE 6:** Average variance extracted (AVE) and reliability of the CFA-model MDS-HSP scale.

Reliability factor	AVE	CR	Cronbach’s alpha
1. Acquiescence to patients’ rights violations	0.702	0.949	0.952
2. Lack of professional competence	0.561	0.920	0.928
3. Disrespect for patients’ autonomy	0.676	0.954	0.957
4. Futile treatment	0.650	0.902	0.892
5. Organizational and social climate	0.676	0.925	0.933
6. Not in patients’ best interest	0.522	0.812	0.832
Total	0.638	0.987	0.974

## 4 Discussion

The objective of this research was to develop a medical distress scale (MDS-HSP Scale) to assess medical distress among medical students and providers in Taiwanese clinical and healthcare settings. The study validated the MDS-HSP Scale’s psychometric qualities using EFA to look at the hidden factor structure and CFA to make sure the dataset matched the model. A preliminary 42-item MDS-HSP Scale was initially developed by the researchers using the EFA. Six factors on this scale accounted for 79.268% of the variance: “acquiescence to patients’ rights violations” (8 items), “lack of professional competence” (9 items), “disrespect for patients’ autonomy” (10 items), “futile treatment” (5 items), “organizational and social climate” (6 items) and “not in patients’ best interest” (4 items).

The EFA findings showed that factor loadings are between 0.961 and 0.527, all higher than Hair et al.’s (2018) proposed threshold of 0.50. By analyzing the mean scores across six distinct factors, the researchers further investigated the diverse aspects of moral distress experienced by healthcare students and providers. Participants had the highest score on the “lack of professional competence” factor (Mean = 4.997 per item: 44.970÷9 = 4.997), followed by the “futile treatment” (Mean = 4.792), “organizational and social climate” (Mean = 4.789), “disrespect for patients’ autonomy” (4.771), and “not in patients’ best interest” (4.707). They gained the lowest scores on the “acquiescence to patients’ rights violations” (Mean = 4.600). This suggests that healthcare students and providers experience the most significant moral distress when they perceive themselves or others as lacking expertise and training to provide appropriate care. This finding corresponds with previous research by Epstein and Hamric (2009) and Oh and Gastmans (2015), indicating that moral distress frequently arises when healthcare providers feel inadequately prepared to manage complex clinical situations.

The second highest mean score was observed in the “futile treatment” factor, indicating significant distress associated with providing treatments perceived as non-beneficial. This is particularly evident in critical and end-of-life care settings, where providers may feel compelled to continue aggressive interventions despite ethical concerns. Hamric et al. (2012) noted that institutional pressures to prolong treatment can intensify moral conflict, especially when such interventions contradict a provider’s professional judgment. Close behind was the “organizational and social climate” factor. This highlights the impact of a negative or unsupportive workplace environment on moral distress. The finding corresponds with Hamric and Epstein’s (2017) study, emphasizing that a lack of open communication, ethical support, and collaborative decision-making within an organization significantly increases the likelihood of moral distress among healthcare providers. Moral distress was also notably associated with “disrespect for patient autonomy” (Mean = 4.771) and “not in patients’ best interest” (Mean = 4.600). Participants reported considerable distress when witnessing actions that undermined ethical principles or disregarded patient autonomy and rights. As Berlinger and Berlinger (2017) noted, excluding patients from decision-making or overriding their preferences can significantly contribute to moral distress. Similarly, some actions may raise moral concerns; however, their ambiguous nature makes it difficult to determine whether they harm patients or go against their best interests. As Johnstone and Hutchinson (2015) pointed out, determining what constitutes a patient’s best interest can be highly subjective, particularly in culturally diverse or ethically complex situations, which may contribute to the comparatively lower distress levels reported in this factor. The lowest mean score was associated with the “acquiescence to patients’ rights violations” factor (Mean = 4.600), still falling within the moderate range of moral distress. This may suggest that healthcare providers often feel powerless when institutional protocols or physician orders conflict with their ethical commitment to uphold patient dignity (Ulrich et al., 2010a).

To rigorously confirm the underlying factor structure, CFA was conducted, yielding the same 42 items with strong factor loadings, in the range of 0.609–0.906, surpassing the benchmark set by Hair et al. (2018). Based on multiple indices, the model’s overall fit was deemed robust and acceptable (Hu and Bentler, 1999). While the EFA-derived MDS-HSP scale already demonstrated acceptable fit indices, the CFA-derived model achieved even better fit. This was evidenced by notable improvements: the TLI and CFI rose by 0.12 and 0.11, respectively, while both the RMSEA and the χ^2^/*df* dropped significantly, by 0.06 and 1.304, respectively (*p* < 0.05). The MDS-HSP scale also met the criteria for convergent validity. Regarding convergent validity, findings indicated that the AVE values for constructs–such as “acquiescence to patients’ rights violations,” “lack of professional competence,” “disrespect for patients’ autonomy,” “futile treatment,” “organizational and social climate,” and “not in patients’ best interest”–were all above the suggested benchmark of 0.50 (Hair et al., 2018; Malhotra, 2008) and lower than their CR scores, exceeding. Therefore, because all AVEs exceeded 0.50 and were lower than their corresponding CRs, which all surpassed 0.60, the scale’s convergent validity is well established. Reliability testing also revealed excellent internal consistency in both the CFA-derived and EFA-derived versions of the scale. Across the full scale and all six factors, both the CR and Cronbach’s alphas ranged from 0.812 to 0.987, well exceeding the accepted minimum of 0.70 (Cunha et al., 2016; Hair et al., 2018), further confirming the scale’s robustness and trustworthiness.

Compared with the MDS and MDS-R, the MDS-HSP demonstrates higher overall reliability, with a Cronbach’s alpha of 0.974 and subscale values ranging from 0.832 to 0.957. By comparison, Corley et al.’s (2001) MDS reported an overall alpha of 0.96, with subscale alphas between 0.82 and 0.97, while Hamric et al.’s (2012) MDS-R showed an alpha of 0.89 for nurses and lower reliability for physicians, ranging from 0.67 in smaller samples to 0.88 in larger cohorts. These results indicate that the MDS-HSP provides robust reliability for measuring moral distress in healthcare settings. The results showed that both EFA and CFA supported the proposed factor structure and demonstrated adequate validity and reliability. Consequently, the scale can serve as a diagnostic and evaluative instrument in educational and clinical settings. Hence, it allows healthcare instructors and administrators to identify which aspects of moral distress are most pronounced and design targeted interventions accordingly. For example, educational interventions such as simulation-based training, clinical skills workshops, and mentorship programs may address distress related to lack of professional competence (Berger, 2014), while institutional policies, organizational cultures, and more straightforward guidelines on end-of-life care can mitigate distress associated with futile treatment (Dzeng et al., 2015; Eagle et al., 2015). Enhancing organizational communication, ethical support, and team-based decision-making may reduce distress linked to negative social climate (Hamric and Epstein, 2017), whereas structured communication, shared decision-making, and patient-centered care can help reduce distress associated with respecting patient autonomy or decisions not in patients’ best interest (Johnstone and Hutchinson, 2015; Zolnierek and DiMatteo, 2009). Reporting mechanisms can also help manage distress related to acquiescence to rights violations (Ulrich et al., 2010b).

Some factors of the MDS-HSP, such as organizational climate and futile treatment, reflect ethical challenges common across healthcare settings internationally and may be generalizable. Other factors–particularly “acquiescence to patients’ rights violations” and perceptions of “disrespect for patients’ autonomy”–are influenced by Taiwan’s cultural context, including hierarchical decision-making, family-centered care, and respect for authority, highlighting the scale’s culturally specific relevance (Ko et al., 2018; Kramer et al., 2002).

Overall, the MDS-HSP provides a framework to connect empirical assessment of moral distress with practical educational and institutional strategies, while acknowledging the cultural differences shaping healthcare providers’ experiences in Taiwan. It will help healthcare instructors and administrators identify ethical challenges and moral distress in clinical practice and design appropriate educational interventions, training programs, or institutional policies to reduce moral distress, thereby facilitating professional well-being and effective care delivery. This recognition can also foster broader cultural understanding of the moral complexities healthcare providers face. Nevertheless, this study has certain limitations. The sample was restricted to Taiwanese participants, limiting the generalizability of the findings to other cultural and clinical contexts. Cultural and contextual factors deeply impact moral distress, and differences in healthcare systems, professional roles, social values, and ethical standards across regions can shape how individuals perceive and respond to such distress. Consequently, the current scale may not fully reflect the complexities of moral distress in settings that differ significantly in cultural, ethical, or socioeconomic conditions. Future research should aim to validate the MDS-HSP scale in diverse healthcare systems and cultural contexts, including adaptations for cross-cultural use. Future research may also focus on longitudinal validation, cross-cultural application, and the effects of targeted interventions on moral distress levels over time.

## 5 Conclusion

This study offers empirical support for the MDS-HSP, confirming its validity and reliability for measuring moral distress in healthcare students and providers within the Taiwanese cultural context. Both exploratory and confirmatory factor analyses demonstrated its strong psychometric properties, establishing the MDS-HSP as a dependable assessment tool. Use of this instrument may guide the development of educational programs, institutional policies, and ethical support frameworks in both healthcare and academic settings.

## Data Availability

The raw data supporting the conclusions of this article will be made available by the authors, without undue reservation.
